# Supplementary material for: Discovery and application of insertion-deletion (INDEL) polymorphisms for QTL mapping of early life-history traits in Atlantic salmon
Source: BMC Genomics. 2010 Mar 8;11:156. doi: 10.1186/1471-2164-11-156 (PMC2838853; doi:10.1186/1471-2164-11-156)
Supplement: Additional file 2 — Information on developed 76 locus single-run INDEL panel in Atlantic salmon. Information on fluorescence labeling, primer concentrations, PCR pooling and links to alignments, INDEL motifs and GENESCAN (Burge and Karlin 1997) predictions of genes/exons are available in html format. [file 1471-2164-11-156-S2.ZIP › Additionalfile2/table8229.html]

Contig Alignment

```
Base	A B C D E F   
1	A . . . . .    
2	G . . . . .    
3	T . . . . .    
4	G . . . . .    
5	C . . . . .    
6	A . . . . .    
7	G . . . . .    
8	C . . . . .    
9	G . . . . .    
10	A . . . . .    
11	G . . . . .    
12	C . . . . .    
13	C . . . . .    
14	A . . . . .    
15	A . . . . .    
16	T . . . . .    
17	G . . . . .    
18	G . . . . .    
19	T . . . . .    
20	T . . . . .    
21	T . . . . .    
22	G . . . . .    
23	G . . . . .    
24	C . . . . .    
25	T . . . . .    
26	G . . . . .    
27	C . . . . .    
28	T . . . . .    
29	C . . . . .    
30	C . . . . .    
31	T . . . . .    
32	T . . . . .    
33	G . . . . .    
34	C . . . . .    
35	A . . . . .    
36	C . . . . .    
37	A . . . . .    
38	G . . . . .    
39	T . . . . .    
40	C . . . . .    
41	A . . . . .    
42	T . . . . .    
43	T . . . . .    
44	A . . . . .    
45	T . . . . .    
46	A . . . . .    
47	A . . . . .    
48	C . . . . .    
49	G . . . . .    
50	T . . . . .    
51	C . . . . .    
52	C . . . . .    
53	T . . . . .    
54	C . . . . .    
55	T . . . . .    
56	A . . . . .    
57	C . . . . .    
58	T . . . . .    
59	A . . . . .    
60	G . . . . .    
61	A . . . . .    
62	G . . . . .    
63	C . . . . .    
64	T . . . . .    
65	T . . . . .    
66	C . . . . .    
67	T . . . . .    
68	A A A A . .    
69	C C C C . .    
70	T T T T . .    
71	C C C C . .    
72	C C C C . .    
73	G G G G . .    
74	G G G G . .    
75	A A A A . .    
76	A A A A . .    
77	A A A A . .    
78	A A A A . .    
79	G G G G . .    
80	G G G G . .    
81	G G G G . .    
82	G G G G . .    
83	A A A A . .    
84	T T T T . .    
85	C C C C . .    
86	C C C C . .    
87	T T T T T .    
88	T T T T T .    
89	G G G G G .    
90	G G G G G .    
91	G G G G G .    
92	A A A A A .    
93	T T T T T .    
94	G G G G G .    
95	C C C C C .    
96	C C C C C .    
97	C C C C C .    
98	C C C C C .    
99	A A A A A .    
100	A A A A A .    
101	C C C C C .    
102	T T T T T .    
103	C C C C C .    
104	T T T T T .    
105	G G G G G .    
106	A A A A A .    
107	C C C C C .    
108	A A A A A .    
109	T T T T T .    
110	C C C C C .    
111	A A A A A .    
112	C C C C C .    
113	C C C C C .    
114	C C C C C .    
115	C C C C C .    
116	A A A A A .    
117	T T T T T .    
118	T T T T T .    
119	G G G G G .    
120	A A A A A .    
121	A A A A A .    
122	G G G G G .    
123	T T T T T .    
124	T T T T T .    
125	G G G G G .    
126	A A A A A .    
127	G G G G G .    
128	G G G G G .    
129	T T T T T .    
130	G G - - G .  2	 indel  
131	A A - - A .  2	 indel  
132	G G - - G .  2	 indel  
133	G G - - G .  2	 indel  
134	T T - - T .  2	 indel  
135	T T T T T .    
136	A A A A A .    
137	G G G G G .    
138	G G G G G .    
139	T T T T T .    
140	T T T T T .    
141	A A A A A .    
142	G G G G G .    
143	G G G G G .    
144	G G G G G .    
145	T T T T T .    
146	T T T T T .    
147	T T T T T .    
148	A A A A A .    
149	G G G G G .    
150	G G G G G .    
151	G G G G G .    
152	T T T T T .    
153	A A A A A .    
154	G G G G G .    
155	G G G G G .    
156	A A A A A .    
157	T T T T T .    
158	C C C C C .    
159	A A A A A .    
160	T T T T T .    
161	C C C C C .    
162	C C N C C .    
163	C C C C C .    
164	A A A A A .    
165	A A A A A .    
166	G G G G G .    
167	G G G G G .    
168	A A A A A .    
169	T T T T T .    
170	C C C C C .    
171	C C C C C .    
172	C C C C C .    
173	G G G G G .    
174	G G G G G .    
175	A A A A A .    
176	T T T T T .    
177	A A A A A .    
178	G G G G G .    
179	T T T T T .    
180	G G G G G .    
181	C C C C C .    
182	A A A A A .    
183	T T T T T .    
184	A A A A A .    
185	A A A A A .    
186	T T T T T .    
187	C C C C C .    
188	C C C C C .    
189	C C C C C .    
190	T T T T T .    
191	T T T T T .    
192	C C C C C .    
193	T T T T T .    
194	A A A A A .    
195	C C C C C .    
196	T T T T T .    
197	C C C C C .    
198	C C C C C .    
199	C C C C C .    
200	T T T T T .    
201	G G G G G .    
202	C C C C C .    
203	A A A A A .    
204	G G G G G .    
205	G G G G G .    
206	A A A A A .    
207	A A A A A .    
208	T T T T T .    
209	G G G G G .    
210	T T T T T .    
211	A A A A A .    
212	A A A A A .    
213	T T T T T .    
214	C C C C C .    
215	T T T T T .    
216	G G G G G .    
217	T T T T T .    
218	A A A A A .    
219	T T T T T .    
220	A A A A A .    
221	T T T T T .    
222	T T T T T .    
223	T T T T T .    
224	C C C C C .    
225	A A A A A .    
226	T T T T T .    
227	T T T T T .    
228	G G G G G .    
229	T T T T T .    
230	T T T T T .    
231	A A A A A .    
232	A A A A A .    
233	T T T T T .    
234	A A A A A .    
235	G G G G G .    
236	T T T T T .    
237	T T T T T .    
238	G G G G G .    
239	G G G G G .    
240	T T T T T .    
241	C C C C C .    
242	C C C C C .    
243	T T T T T .    
244	T T T T T .    
245	T T T T T .    
246	T T T T T .    
247	T T T T T .    
248	T T T T T .    
249	A A A A A .    
250	A A A A A .    
251	G G G G G .    
252	A A A A A .    
253	A A A A A .    
254	T T T T T .    
255	T T T T T .    
256	T T T T T .    
257	G G G G G .    
258	A A A A A .    
259	T T T T T .    
260	T T T T T .    
261	T T T T T .    
262	T T T T T .    
263	A A A A A .    
264	T T T T T .    
265	C C C C C .    
266	A A A A A .    
267	G G G G G .    
268	T T T T T .    
269	G G G G G .    
270	T T T T T .    
271	T T T T T .    
272	G G G G G .    
273	C C C C C .    
274	T T T T T .    
275	T T T T T .    
276	T T T T T .    
277	T T T T T .    
278	G G G G G .    
279	A A A A A .    
280	A A A A A .    
281	A A A A A .    
282	A A A A A .    
283	T T T T T .    
284	A A A A A .    
285	G G G G G .    
286	G G G G G .    
287	G G G G G .    
288	T T T T T .    
289	G G G G G .    
290	C C C C C .    
291	A A A A A .    
292	C C C C C .    
293	C C C C C .    
294	T T T T T .    
295	T T T T T .    
296	G G G G G .    
297	T T T T T .    
298	C C C C C .    
299	A A A A A .    
300	A A A A A .    
301	T T T T T .    
302	A A A A A .    
303	T T T T T .    
304	T T T T T .    
305	T T T T T .    
306	C C C C C .    
307	T T T T T .    
308	A A A A A .    
309	T T T T T .    
310	T T T T T .    
311	A A A A A .    
312	T T T T T .    
313	T T T T T .    
314	T T T T T .    
315	G G G G G .    
316	T T T T T .    
317	A A A A A .    
318	T T T T T .    
319	T T T T T .    
320	A A A A A .    
321	C C C C C .    
322	C C C C C .    
323	C C C C C .    
324	A A A A A .    
325	T T T T T .    
326	T T T T T .    
327	G G G G G .    
328	T T T T T .    
329	A A A A A .    
330	A A A A A .    
331	G G G G G .    
332	C C C C C .    
333	G G G G G G    
334	T T T T T T    
335	A A A A A A    
336	A A A A A A    
337	T T T T T T    
338	T T T T T T    
339	A A A A A A    
340	T T T T T T    
341	G G G G G G    
342	T T T T T T    
343	T T T T T T    
344	T T T T T T    
345	G G G G G G    
346	A A A A A A    
347	C C C C C C    
348	T T T T T T    
349	C C C C C C    
350	T T T T T T    
351	T T T T T T    
352	A A A A A A    
353	C C C C C C    
354	A A A A A A    
355	A A A A A A    
356	T T T T T T    
357	T T T T T T    
358	T T T T T T    
359	T T T T T T    
360	T T T T T T    
361	A A A A A A    
362	G G G G G G    
363	T T T T T T    
364	T T T T T T    
365	G G G G G G    
366	A A A A A A    
367	G G G G G G    
368	A A A A A A    
369	A A A A A A    
370	T T T T T T    
371	A A A A A A    
372	A A A A A A    
373	T T T T T T    
374	G G G G G G    
375	A A A A A A    
376	C C C C C C    
377	A A A A A A    
378	A A A A A A    
379	A A A A A A    
380	T T T T T T    
381	G G G G G G    
382	A A A A A A    
383	T T T T T T    
384	G G G G G G    
385	C C C C C C    
386	C C C C C C    
387	T T T T T T    
388	G G G G G G    
389	T T T T T T    
390	A A A A A A    
391	A A A A A A    
392	A A A A A A    
393	T T T T T T    
394	G G G G G G    
395	A A A A A A    
396	A A A A A A    
397	A A A A A A    
398	T T T T T T    
399	G G G G G G    
400	T T T T T T    
401	G G G G G G    
402	T T T T T T    
403	G G G G G G    
404	T T T T T T    
405	T T T T T T    
406	G G G G G G    
407	C C C C C C    
408	A A A A A A    
409	C C C C C C    
410	A A A A A A    
411	A A A A A A    
412	G G G G G G    
413	T T T T T T    
414	C C C C C C    
415	C C C C C C    
416	T T T T T T    
417	C C C C C C    
418	A A A A A A    
419	C C C C C C    
420	A A A A A A    
421	A A A A A A    
422	T T T T T T    
423	T T T T T T    
424	T T T T T T    
425	G G G G G G    
426	T T T T T T    
427	G G G G G G    
428	G G G G G G    
429	A A A A A A    
430	A A A A A A    
431	G G G G G G    
432	C C C C C C    
433	A A A A A A    
434	A A A A A A    
435	C C C C C C    
436	T T T T T T    
437	A A A A A A    
438	G G G G G G    
439	A A A A A A    
440	T T T T T T    
441	G G G G G G    
442	T T T T T T    
443	G G G G G G    
444	G G G G G G    
445	A A A A A A    
446	A A A A A A    
447	G G G G G G    
448	C C C C C C    
449	A A A A A A    
450	A A A A A A    
451	C C C C C C    
452	T T T T T T    
453	A A A A A A    
454	G G G G G G    
455	A A A A A A    
456	T T T T T T    
457	G G G G G G    
458	G G G G G G    
459	G G G G G G    
460	G G G G G G    
461	T T T T T T    
462	A A A A A A    
463	G G G G G G    
464	C C C C C C    
465	T T T T T T    
466	A A A A A A    
467	T T T T T T    
468	A A A A A A    
469	A A A A A A    
470	A A A A A A    
471	T T T T T T    
472	T T T T T T    
473	T T T T T T    
474	G G G G G G    
475	T T T T T T    
476	T T T T T T    
477	T T T T T T    
478	T T T T T T    
479	G G G G G G    
480	T T T T T T    
481	G G G G G G    
482	T T T T T T    
483	G G G G G G    
484	G G G G G G    
485	A A A A A A    
486	C C C C C C    
487	C C C C C C    
488	A A A A A A    
489	G G G G G G    
490	A A A A A A    
491	A A A A A A    
492	A A A A A A    
493	G G G G G G    
494	T T T T T T    
495	C C C C C C    
496	T T T T T T    
497	A A A A A A    
498	A A A A A A    
499	A A A A A A    
500	T T T T T T    
501	G G G G G G    
502	T T T T T T    
503	A A A A A A    
504	C C C C C C    
505	A A A A A A    
506	A A A A A A    
507	A A A A A A    
508	A A A A A A    
509	A A A A A A    
510	A A A A A A    
511	T T T T T T    
512	G G G G G G    
513	A A A A A A    
514	T T T T T T    
515	T T T T T T    
516	T T T T T T    
517	C C C C C C    
518	A A A A A A    
519	T T T T T T    
520	G G G G G G    
521	A A A A A A    
522	T T T T T T    
523	C C C C C C    
524	C C C C C C    
525	T T T T T T    
526	A A A A A A    
527	T T T T T T    
528	T T T T T T    
529	T T T T T T    
530	A A A A A A    
531	T T T T T T    
532	T T T T T T    
533	T T T T T T    
534	T T T T T T    
535	T T T T T T    
536	A A A A A A    
537	A A A A A A    
538	G G G G G G    
539	A A A A A A    
540	T T T T T T    
541	A A A A A A    
542	A A A A A A    
543	T T T T T T    
544	T T T T T T    
545	A A A A A A    
546	G G G G G G    
547	T T T T T T    
548	T T T T T T    
549	A A A A A A    
550	C C C C C C    
551	T T T T T T    
552	G G G G G G    
553	C C C C C C    
554	A A A A A A    
555	A A A A A A    
556	G G G G G G    
557	A A A A A A    
558	T T T T T T    
559	A A A A A A    
560	G G G G G G    
561	G G G G G G    
562	A A A A A A    
563	C C C C C C    
564	T T T T T T    
565	G G G G G G    
566	C C C C C C    
567	A A A A A A    
568	A A A A A A    
569	T T T T T T    
570	G G G G G G    
571	T T T T T T    
572	A A A A A A    
573	T T T T T T    
574	T T T T T T    
575	T T T T T T    
576	T T T T T T    
577	T T T T T T    
578	A A A A A A    
579	T T T T T T    
580	C C C C C C    
581	A A A A A A    
582	A A A A A A    
583	A A A A A A    
584	A A A A A A    
585	A A A A A A    
586	G G G G G G    
587	C C C C C C    
588	T T T T T T    
589	T T T T T T    
590	T T T T T T    
591	T T T T T T    
592	C C C C C C    
593	A A A A A A    
594	G G G G G G    
595	T T T T T T    
596	G G G G G G    
597	C C C C C C    
598	A A A A A A    
599	T T T T T T    
600	T T T T T T    
601	T T T T T T    
602	T T T T T T    
603	G G G G G G    
604	G G G G G G    
605	A A A A A A    
606	A A A A A A    
607	A A A A A A    
608	C C C C C C    
609	A A A A A A    
610	T T T T T T    
611	C C C C C C    
612	T T T T T T    
613	T T T T T T    
614	G G G G G G    
615	G G G G G G    
616	G G G G G G    
617	A A A A A A    
618	C C C C C C    
619	A A A A A A    
620	G G G G G G    
621	T T T T T T    
622	A A A A A A    
623	C C C C C C    
624	A A A A A A    
625	G G G G G G    
626	A A A A A A    
627	T T T T T T    
628	T T T T T T    
629	T T T T T T    
630	T T T T T T    
631	G G G G G G    
632	T T T T T T    
633	T T T T T T    
634	T T T T T T    
635	T T T T T T    
636	T T T T T T    
637	C C C C C C    
638	A A A A A A    
639	T T T T T T    
640	T T T T T T    
641	T T T T T T    
642	G G G G G G    
643	T T T T T T    
644	T T T T T T    
645	T T T T T T    
646	T T T T T T    
647	T T T T T T    
648	G G G G G G    
649	T T T T T T    
650	C C C C C C    
651	G G G G G G    
652	C C C C C C    
653	T T T T T T    
654	G G G G G G    
655	C C C C C C    
656	T T T T T T    
657	G G G G G G    
658	T T T T T T    
659	C C C C C C    
660	A A A A A A    
661	T T T T T T    
662	C C C C C C    
663	C C C C C C    
664	T T T T T T    
665	G G G G G G    
666	A A A A A A    
667	G G G G G G    
668	G G G G G G    
669	T T T T T T    
670	A A A A A A    
671	G G G G G G    
672	C C C C C C    
673	T T T T T T    
674	A A A A A A    
675	A A A A A A    
676	A A A A A A    
677	C C C C C C    
678	C C C C C C    
679	G G G G G G    
680	C C C C C C    
681	A A A A A A    
682	T T T T T T    
683	T T T T T T    
684	T T T T T T    
685	A A A A A A    
686	G G G G G G    
687	A A A A A A    
688	A A A A A A    
689	T T T T T T    
690	A A A A A A    
691	T T T T T T    
692	A A A A A A    
693	. T T T T T    
694	. T T T T T    
695	. C C C C C    
696	. C C C C C    
697	. T T T T T    
698	. A A A A A    
699	. T A A A A    
700	. . A A A A    
701	. . G G G G    
702	. . A A A A    
703	. . A A A A    
704	. . T T T T    
705	. . T T T T    
706	. . A A A A    
707	. . C C C C    
708	. . A A A A    
709	. . T T T T    
710	. . T T T T    
711	. . T T T T    
712	. . T T T T    
713	. . T T T T    
714	. . A A A A    
715	. . G G G G    
716	. . A A A A    
717	. . G G G G    
718	. . C C C C    
719	. . T T T T    
720	. . T T T T    
721	. . T T T T    
722	. . T T T T    
723	. . G G G G    
724	. . A A A A    
725	. . T T T T    
726	. . G G G G    
727	. . C C C C    
728	. . A A A A    
729	. . T T T T    
730	. . G G G G    
731	. . C C C C    
732	. . G G G G    
733	. . A A A A    
734	. . T T T T    
735	. . A A A A    
736	. . T T T T    
737	. . T T T T    
738	. . T T T T    
739	. . T T T T    
740	. . A A A A    
741	. . C C C C    
742	. . C C C C    
743	. . T T T T    
744	. . . A A A    
745	. . . A A A    
746	. . . T T T    
747	. . . A A A    
748	. . . A A A    
749	. . . A A A    
750	. . . G G G    
751	. . . A A A    
752	. . . C C C    
753	. . . T T T    
754	. . . A A A    
755	. . . T T T    
756	. . . T T T    
757	. . . G G G    
758	. . . G G G    
759	. . . T T T    
760	. . . T T T    
761	. . . T T T    
762	. . . T T T    
763	. . . T T T    
764	. . . T T T    
765	. . . A A A    
766	. . . T T T    
767	. . . T T C    
768	. . . T T .
```
